# 655. Patterns of Interferon-Gamma Release Assay (IGRA) Testing for Tuberculosis in Patients Less Than 2 Years Old

**DOI:** 10.1093/ofid/ofab466.852

**Published:** 2021-12-04

**Authors:** Mary Tabatneck, Jeffrey Campbell, Mingwei Sun, Wei He, Gabriella S Lamb, Gabriella S Lamb, Don Goldmann, Vishakha Sabharwal, Thomas Sandora, Jessica Haberer

**Affiliations:** 1 Boston Children's Hospital, Boston, MA; 2 Massachusetts General Hospital, Boston, MA; 3 Boston Children's Hospital, Harvard Medical School, Washington, DC; 4 MD, Boston, MA

## Abstract

**Background:**

The American Academy of Pediatrics recommends use of Interferon-Gamma Release Assays (IGRAs) to diagnose tuberculosis (TB) infection in patients ≥2 years old. However, IGRAs are not currently recommended in younger patients due to limited data and concerns of invalid/indeterminate test results, which occur if there is a positive or negative control failure. We sought to characterize the patterns of IGRA use in clinical practice and results of IGRAs in patients < 2 years old.

**Methods:**

We conducted a retrospective cohort study of children < 2 years old at two large health systems in the Boston area who had IGRA and/or tuberculin skin test (TST) performed from October 1, 2015 – January 31, 2021. We reviewed medical records to determine IGRA test type, IGRA result (positive, negative, invalid/indeterminate) and location of testing (outpatient primary care, outpatient subspecialty, inpatient). We summarized test interpretability, location, and changes in proportion of IGRA vs. TST.

**Results:**

We identified 330 IGRA (268 T-SPOT.TB, 62 QuantiFERON Gold) and 2029 TST results among 1982 patients who were < 2 years old (range: 11 days – 1.9 years). Monthly proportion of IGRAs among all TB tests ordered increased from 2015 to 2021 (Figure 1) (Pearson correlation coefficient 0.85, P < 0.001). Among IGRA results, 314 (95%) were negative, 3 (1%) were positive, and 13 (4%) were invalid/indeterminate (11 T-SPOT.TB, 2 QuantiFERON Gold). Of 324 IGRA tests for which testing location was known, 233 (72%) and 91 (28%) were ordered in outpatient and inpatient settings, respectively. Of tests in outpatient settings, 132 (57%) were ordered in primary care offices, 53 (23%) were ordered in subspecialist offices, and 48 (21%) were obtained in outpatient labs of unidentified clinics.

Tuberculosis infection tests and proportion IGRA.

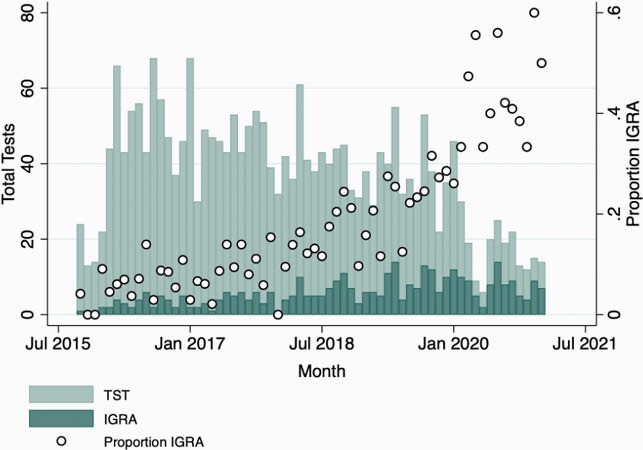

Total number of tests and proportion of IGRA:TST obtained by month, from October 2015-January 2021.

**Conclusion:**

While most TB infection tests in this age group were TSTs, the monthly proportion of tests that were IGRAs increased over time between 2015-2021. IGRAs were obtained in varied clinical settings. In this low-burden setting, rates of invalid/indeterminate IGRAs were low among children < 2 years old, which suggests that IGRAs are reasonable TB testing options for patients < 2 years old, and may be preferred given limitations of TSTs.

**Disclosures:**

**Gabriella S. Lamb, MD, MPH**, Nothing to disclose

